# Efficacy and Safety of Atezolizumab as a PD-L1 Inhibitor in the Treatment of Cervical Cancer: A Systematic Review

**DOI:** 10.3390/biomedicines12061291

**Published:** 2024-06-11

**Authors:** Milan Daniel Velimirovici, Catalin Vladut Ionut Feier, Razvan Constantin Vonica, Alaviana Monique Faur, Calin Muntean

**Affiliations:** 1 Doctoral School, “Victor Babes” University of Medicine and Pharmacy, 2 E. Murgu Sq., 300041 Timisoara, Romania; milan.velimirovici@umft.ro; 2 First Discipline of Surgery, Department X-Surgery, “Victor Babes” University of Medicine and Pharmacy, 2 E. Murgu Sq., 300041 Timisoara, Romania; catalin.feier@umft.ro; 3 First Surgery Clinic, “Pius Brinzeu” Clinical Emergency Hospital, 300723 Timisoara, Romania; 4 Preclinical Department, Discipline of Physiology, Faculty of General Medicine, “Lucian Blaga” University of Sibiu, 550169 Sibiu, Romania; 5 Faculty of Medicine, Victor Babes” University of Medicine and Pharmacy, 2 E. Murgu Sq., 300041 Timisoara, Romania; alaviana.faur@student.umft.ro; 6 Medical Informatics and Biostatistics, Department III-Functional Sciences, “Victor Babes” University of Medicine and Pharmacy, 2 E. Murgu Sq., 300041 Timisoara, Romania; cmuntean@umft.ro

**Keywords:** oncology, gynecology, cervical cancer, systematic review

## Abstract

Background and objectives: The efficacy and safety of PD-L1 inhibitors in the treatment of cervical cancer is an ongoing research question. This review aims to establish a clear profile of atezolizumab, examining its impact on survival outcomes, response rates, and safety measured by serious adverse events (SAEs). Materials and methods: A literature search was conducted using PubMed, Scopus, and Web of Science, focusing on articles published up to February 2024. The review followed the PRISMA guidelines and synthesized outcomes from four randomized trial studies involving atezolizumab administered at 1200 mg IV every three weeks, alone or in combination with chemoradiotherapy. Results: A total of 284 patients received atezolizumab, the majority being advanced stage cervical cancer (IVA-IVB). Median follow-up times ranged from 9 weeks to 32.9 months. It was found that combining atezolizumab with standard therapies extended median progression-free survival (PFS) from 10.4 to 13.7 months and overall survival (OS) from 22.8 to 32.1 months, according to the phase III trial. Monotherapy and initial treatment settings with atezolizumab also showed promising efficacy, with disease-free survival rates at 24 months reaching 79% compared to 52% with standard therapy alone. However, the treatment was associated with high rates of SAEs, reaching up to 79% in more intensive treatment combinations. Conclusions: Atezolizumab demonstrates significant potential in improving PFS and OS in patients with cervical cancer, supporting its inclusion as a first-line treatment option. Despite the efficacy benefits, the high incidence of SAEs necessitates careful patient selection and management strategies to mitigate risks. This systematic review supports the continued evaluation of atezolizumab in broader clinical trials to refine its therapeutic profile and safety measures in the context of cervical cancer treatment.

## 1. Introduction

Cervical cancer is the fourth most common cancer among women worldwide, with approximately 600,000 new cases in 2022, and up to 350,000 deaths, according to the World Health Organization (WHO) [1,2]. Persistent infection with high-risk human papillomavirus (HPV) types, primarily HPV-16 and HPV-18, is identified as the major cause [3,4,5]. The global burden remains high despite the implementation of preventive HPV vaccinations and screening programs, particularly in regions with limited access to these preventive measures [6,7].

Standard treatments for cervical cancer include surgery, radiotherapy, and platinum-based chemotherapy [8,9]. Advanced and recurrent cases are less responsive to these interventions, showing five-year survival rates of about 17% for metastatic disease, according to the recent statistics [10,11]. The introduction of targeted therapies and immune checkpoint inhibitors presents new avenues for improving outcomes in these advanced stages [12]. However, accurate disease progression remains difficult, as the clinical evolution of the patient does not always correlate with the progression of disease at a molecular and microscopic level. Therefore, the importance of histopathology in the diagnosis, classification and post-treatment evaluation of cervical cancer underscores a critical dimension of research, particularly in evaluating the efficacy of new treatments like atezolizumab. Advanced imaging and classification techniques, such as those utilizing LASSO and EL-SVM for biopsy image classification or integrating deep convolutional features through AF-SENet or ViT-AMC networks, that help contribute significantly to the precision and interpretability of diagnoses [13,14]. These methods not only enhance the accuracy of tumor grading but also enable a more nuanced understanding of tumor behavior and pathology, which is essential for tailoring treatment strategies. By comparing the outcomes and safety profiles of atezolizumab with these detailed histopathological insights, this review aims to bridge the gap between macro-level treatment effects and micro-level cellular responses. This approach will provide a more comprehensive evaluation of atezolizumab’s potential, assessing its role not only in extending survival but also in aligning with precise histopathological classifications to optimize patient outcomes.

PD-1 and PD-L1 inhibitors have shown efficacy in several solid tumors, leading to improved survival outcomes [15]. For example, pembrolizumab has been approved for PD-L1-positive cervical cancer based on improved overall survival and progression-free survival rates compared to chemotherapy in clinical trials like KEYNOTE-158 [16]. Atezolizumab specifically has been evaluated in several phase I and II trials for PD-L1-positive cancers [16,17]. Data indicated that atezolizumab provided a complete response of 6% in solid cancers and a partial response rate of 16% with a median progression free survival (PFS) between 1.5 and 6 months [18], highlighting its potential as a viable option for patients with advanced cervical cancer refractory to standard treatments.

This systematic review aims to critically analyze the role of atezolizumab in cervical cancer treatment, hypothesizing that it offers superior efficacy and safety profiles compared to conventional therapies. The review will aggregate findings from various clinical trials, focusing on objective response rates, median survival times, safety profiles, and quality of life outcomes, aiming to establish a clear therapeutic profile of atezolizumab for cervical cancer. This study is motivated by the need to improve treatment precision for advanced cervical cancer, where traditional therapies often fall short. By integrating advanced histopathological techniques and evaluating the efficacy of atezolizumab, a PD-L1 inhibitor, this research aims to bridge the gap between macroscopic treatment effects and microscopic cellular responses. This approach seeks to enhance patient outcomes by tailoring therapies to more precisely match the tumor’s molecular profile.

## 2. Materials and Methods

### 2.1. Eligibility Criteria

**Figure 1:**
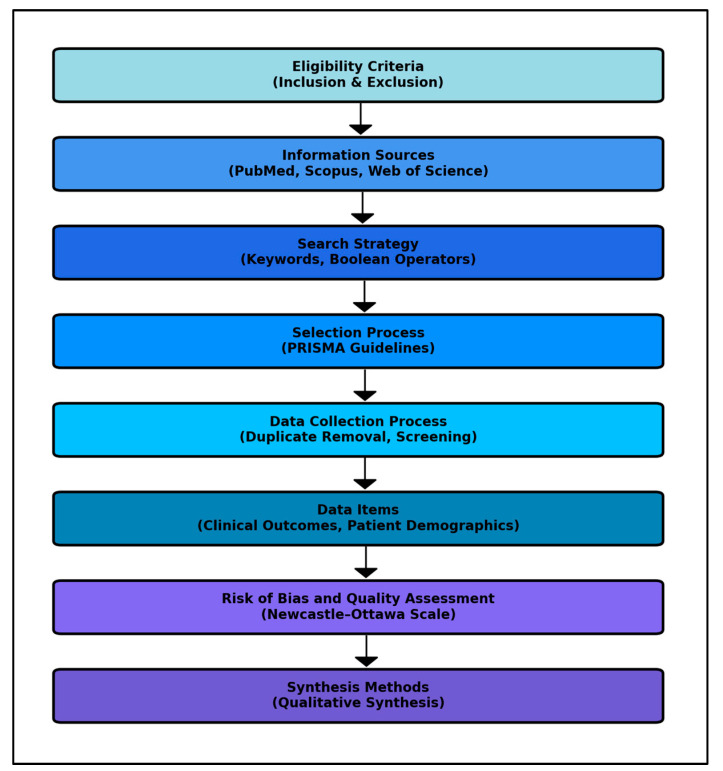
Materials and methods flowchart.

The review considered studies for the final analysis based on the following inclusion criteria and based on the methodology described in Figure 1 flowchart: (1) studies must involve patients diagnosed with cervical cancer, including patients across all age groups and both genders, where Atezolizumab is used as a treatment option either as a monotherapy or in combination with other therapeutic agents; (2) research must explicitly examine the clinical outcomes following the use of Atezolizumab, with particular emphasis on overall survival, progression-free survival, and safety profiles or side effects; (3) the review will include a broad array of study designs, such as randomized controlled trials, observational studies, clinical trials, cohort studies, case–control studies, and cross-sectional studies. Both prospective and retrospective studies will be considered to capture a comprehensive view of Atezolizumab’s impact; (4) studies that utilize validated instruments or clearly defined parameters to assess survival rates, molecular markers of response, adverse events, and quality of life outcomes; and (5) only peer-reviewed articles published in English will be included to ensure the feasibility of thorough review and analysis.

The exclusion criteria include (1) research not involving human participants, such as in vitro or animal model studies related to cervical cancer, will be excluded to focus solely on human patient experiences and outcomes; (2) studies not specifically examining patients with cervical cancer or those that do not differentiate the impact of Atezolizumab on this specific patient group will be excluded, or used other different ICIs; (3) studies that do not provide clear, quantifiable outcomes related to survival rates, molecular responsiveness, or lack sufficient detail for a comprehensive analysis will be excluded; (4) to maintain the credibility and reliability of the data included in the review, grey literature, including non-peer-reviewed articles, preprints, conference proceedings, general reviews, commentaries, and editorials, will be excluded; and (5) low quality studies assessed by quantifiable methods that can determine significant discrepancies in reported data will also be excluded. 

### 2.2. Information Sources

This study adopted an extensive search strategy across three electronic databases, including PubMed, Scopus, and Web of Science. The literature search was targeted to include publications up to 23 February 2024, as the initial search date, ensuring the inclusion of the most recent and relevant studies on the topic. The primary objective of the search strategy was to collect studies that evaluate the clinical outcomes, molecular characteristics, patient demographics, and treatment modalities associated with the use of Atezolizumab in treating metastatic cervical cancer. 

### 2.3. Search Strategy

The search strategy utilizes an expansive array of keywords and phrases pertinent to the study’s objectives, focusing on the nuances of immune checkpoint inhibition and clinical outcomes in metastatic cervical cancer. Key search terms include “cervical cancer”, “metastatic cervical cancer”, “metastatic disease”, “advanced cervical cancer”, “Atezolizumab”, “immune checkpoint inhibitors”, “PD-1 inhibitors”, “PD-L1 expression”, “clinical efficacy”, “molecular insights”, “survival outcomes”, “progression-free survival”, “overall survival”, “treatment response”, “adverse effects”, “immunotherapy”, “cancer immunotherapy”, “patient demographics”, “treatment modalities”, and “biomarkers.”

To ensure a comprehensive and efficient literature retrieval, Boolean operators (AND, OR, NOT) were employed to effectively combine and refine these terms along with relevant Medical Subject Headings (MeSH) and keywords. The search string included the following: ((“cervical cancer” OR “advanced cancer” OR “metastatic cervical cancer”) AND (“Atezolizumab” OR “immune checkpoint inhibitors” OR “PD-1 inhibitors”) AND (“clinical efficacy” OR “survival outcomes” OR “treatment response”) AND (“molecular insights” OR “PD-L1 expression” OR “biomarkers”) AND (“overall survival” OR “progression-free survival”) AND (“adverse effects” OR “patient demographics”)).

### 2.4. Selection Process

In accordance with the Preferred Reporting Items for Systematic Reviews and Meta-Analyses (PRISMA) guidelines [19], our selection process involved a structured and transparent method to ensure the reproducibility of our research. Initially, all retrieved records were independently screened by two reviewers to determine their eligibility based on the predefined inclusion and exclusion criteria. Discrepancies between reviewers were resolved through discussion or, if necessary, consultation with a third reviewer. The review protocol and its detailed selection methodology have been registered and are openly accessible on the Open Science Framework (OSF) with the registration code osf.io/xckb9, ensuring transparency of our research process and findings.

### 2.5. Data Collection Process

The data collection process for this systematic review commenced with the removal of duplicate entries, followed by a rigorous screening of abstracts by two independent reviewers (C.V.I.F. and C.M.) to assess each study’s relevance based on predefined inclusion and exclusion criteria. Discrepancies between reviewers were resolved through discussion or, if necessary, consultation with a third reviewer to achieve consensus. The initial database search yielded the total number of articles which were evaluated and identified for inclusion in the final study.

### 2.6. Data Items

For this systematic review, we collected data on key clinical outcomes related to Pembrolizumab treatment in cervical cancer as defined by National Comprehensive Cancer Network (NCCN) guidelines [20]. The primary outcomes included overall survival (OS) and progression-free survival at 1 year, 3 years, and 5 years, along with adverse event rates, as these metrics are critical in evaluating the efficacy and safety of cancer therapies (NCCN Guidelines for Cervical Cancer, 2023). Secondary outcomes were aimed to focus on response rates and duration of response, utilizing Response Evaluation Criteria in Solid Tumors (RECIST) for assessment standardization [21].

Study and patient characteristics data were also collected, encompassing study design, geographic location, patient demographics (age, gender), and treatment details (dosing, combination therapies). Biomarker data, particularly PD-L1 expression, were included due to its predictive value in response to Atezolizumab, following FDA guidance on biomarker inclusion in clinical trials.

Metastatic cervical cancer was defined as per the American Joint Committee on Cancer (AJCC) staging system, focusing on cases where cancer has spread to distant sites, relevant for assessing Atezolizumab’s role in advanced disease stages (AJCC Cancer Staging Manual, 8th Edition) [22].

The administration of Atezolizumab was characterized according to its labeled use in the treatment of metastatic cervical cancer and depending on the study protocol of the clinical trials involved, reflecting standard practices outlined in the latest FDA drug approval announcements for oncology drugs.

### 2.7. Risk of Bias and Quality Assessment

For the systematic assessment of study quality and determination of risk of bias within the included studies, our review employed a dual approach, integrating both qualitative and quantitative evaluation methods. Initially, the quality of observational studies was evaluated using the Newcastle–Ottawa Scale [23], a widely recognized tool that assesses three critical dimensions: the selection of study groups, the comparability of these groups, and the ascertainment of either the exposure or outcome of interest for case–control or cohort studies, respectively. Each study is awarded stars in these categories, cumulating in a score that classifies the study quality as either low, medium, or high. To ensure the objectivity and reproducibility of our quality assessment process, each study was independently evaluated by two researchers. Discrepancies in quality assessment scores were resolved through discussion, or if necessary, consultation with a third researcher.

### 2.8. Synthesis Methods

In this systematic review, we integrated findings from selected studies through a qualitative synthesis, given the variability in study designs and outcome measures reported. The selection of studies for synthesis was based on their alignment with predefined inclusion criteria, focusing on Atezolizumab and its impact on molecular mechanisms involved in potentially improved survival rates, morbidity, and mortality. To prepare data for synthesis, we performed a tabulation of survival outcomes, surgical success rates, and complication rates, while handling missing data by noting their absence and acknowledging potential impacts on our findings. Results from individual studies were summarized and presented descriptively, comparing survival outcomes and treatment effectiveness across diverse geographic and clinical settings. 

A meta-analysis was performed to evaluate the efficacy of atezolizumab in treating cervical cancer in terms of progression-free survival and overall survival. Heterogeneity among study results was quantified using the I² statistic, which describes the percentage of total variation across studies that is due to heterogeneity rather than chance. A high I^2^ value indicates substantial variability among the studies. All analyses were performed using standard statistical software, ensuring that all estimates were accompanied by 95% confidence intervals to assess the precision of the findings.

## 3. Results

### 3.1. Study Selection and Study Characteristics

**Figure 2:**
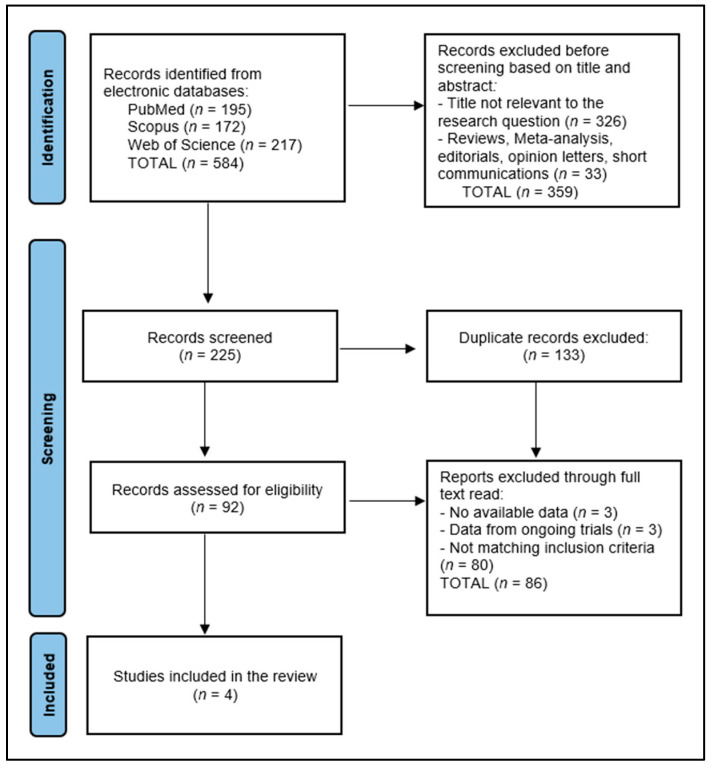
PRISMA flow diagram.

A total of 584 articles were identified according to the initial search, of which 133 duplicate entries were eliminated, 359 records excluded before screening based on title and abstract, and 86 articles excluded after full read for not matching the inclusion criteria or having no available data. The systematic review included a total of four studies in the final analysis, delineated in Figure 2, spanning a period from 2020 to 2024.

The four included studies [24,25,26,27] were conducted internationally between 2020 and 2024. These studies, detailed by Friedman et al. [24], Tabernero et al. [25], Mayadev et al. [26], and Oaknin et al. [27], were all designed as randomized clinical trial designs, with phases ranging from I/Ib to III [27]. Each study was assessed as high quality, as described in Table 1. 

### 3.2. Results of Individual Studies

The clinical trials encompassed a total number of 284 patients who received Atezolizumab treatment. Friedman et al. [24] included the smallest cohort with 11 patients, where the median age was 48 years. This study employed a combination of atezolizumab and bevacizumab, but did not report the time since diagnosis, focusing instead on immediate treatment responses. The performance status was relatively high, with a majority (64%) of patients classified under ECOG 0, indicating full functionality.

In contrast, Tabernero et al. [25] enrolled a larger group of 27 patients, with a median age of 49 years. This study uniquely lacked a comparison group, focusing solely on atezolizumab’s outcomes. The performance statuses indicated a lesser degree of patient functionality compared to Friedman et al., with a majority (59.3%) at ECOG 1. Detailed duration since diagnosis and metastasis provided insights into the disease progression timeline, averaging 3.47 years since diagnosis and 1.52 years since metastasis.

Mayadev et al. [26] presented data from 40 patients, all newly diagnosed, which allowed for an assessment of atezolizumab combined with standard chemoradiation from the onset of treatment. The uniformity in performance status (ECOG <2: 100%) indicated a selected patient group with relatively good physical functioning.

Oaknin et al. [27], in the largest and most complex study, involved 206 patients and provided a direct comparison between atezolizumab combined with bevacizumab and chemotherapy and a control group receiving only the latter two treatments. The median age was slightly higher at 51 years. This study was also significant for its detailed breakdown of disease status at screening, revealing a majority with recurrent disease (73%), alongside newly diagnosed stage IVB (21%) and persistent disease (6%). The performance status mirrored that of a well-functioning cohort similar to Friedman et al. [24], with two-thirds of the patients at ECOG 0, as presented in Table 2.

### 3.3. Results of Synthesis

The reviewed studies [24,25,26,27] documented in Table 3 offer a detailed look into the disease characteristics of cervical cancer patients treated with atezolizumab, revealing a diverse array of stages, histological types, HPV/PD-L1 statuses, previous treatments, and rates of serious adverse events.

Friedman et al. [24] examined patients exclusively at advanced stages (IVA-IVB), with a fairly even distribution between squamous cell carcinoma (SCC) and adenocarcinoma (ACC). Most patients had undergone prior pelvic radiation combined with cisplatin and had been treated according to the GOG 240 regimen. Despite this extensive pretreatment, the occurrence of SAEs of grade 3 or higher was relatively low at 18.1%, suggesting a manageable safety profile in a heavily pretreated population.

In contrast, the study by Tabernero et al. [25] presented a cohort with a predominant IVB stage (96.3%), with SCC slightly more prevalent than ACC. A high percentage of patients (77.8%) were PD-L1 positive, indicating a selected subgroup likely to respond to PD-L1 inhibition. Patients in this study had significant exposure to prior treatments, including radiotherapy and surgery, yet the overall incidence of SAEs was modest at 3.6%, highlighting the potential tolerability of atezolizumab in this setting.

Mayadev et al. [26] focused on a specific patient group with stage IIB cervical cancer, where prior treatment primarily consisted of brachytherapy (77.5%). The rate of SAEs was higher at 25%, which could reflect the intensity of prior local treatments impacting overall tolerability. Oaknin et al. [27], in the largest study, covered a broader spectrum of disease stages from early (I) to advanced (IVB), predominantly featuring SCC (80%). This study also had a diverse range of prior treatments including surgery and chemotherapy. Notably, it reported the highest rate of SAEs at 79%, which might be associated with the complexity of cases handled, including patients at various stages of advanced disease, and possibly more aggressive disease features necessitating intensive treatments (Table 3).

Friedman et al. [24] investigated the combination of atezolizumab with bevacizumab, administered every three weeks. Despite a substantial duration of follow-up (median 9 weeks), the study failed to meet its efficacy endpoints. The objective response rate (ORR) was 0%, with a disease control rate (DCR) of 60%. The median progression-free survival (PFS) was relatively short at 2.9 months, and the median overall survival (OS) was 8.9 months. This study concluded that the combination did not achieve significant response rates in the evaluated patients, and the survival outcomes were modest, indicating limited clinical benefit in this setting.

Tabernero et al. [25] focused on atezolizumab monotherapy, allowing treatment until clinical benefit was lost or unacceptable toxicity occurred. Over a follow-up of 18 weeks, the study reported a PFS rate of 44.4% and an ORR of 14.8%. These outcomes suggest that atezolizumab alone can be effective for some patients with cervical cancer, offering a potential treatment option where traditional therapies may not be suitable.

Mayadev et al. [26] explored the use of atezolizumab in a concurrent setting with chemoradiotherapy in newly diagnosed patients, finding that the drug demonstrated immune-modulating activity and was safe to use. Over a median follow-up of 20 months, the two-year disease-free survival (DFS) was significantly higher in the atezolizumab group (79%) compared to those receiving only chemoradiotherapy (52%). This significant improvement in DFS highlights the potential of integrating atezolizumab into the initial treatment phase for enhancing long-term outcomes.

Lastly, Oaknin et al. [27] presented data from a large study where atezolizumab was added to a standard regimen of bevacizumab and chemotherapy. Over a substantial median follow-up of 32.9 months, the addition of atezolizumab extended median PFS from 10.4 months in the standard therapy group to 13.7 months in the treatment group, and median OS from 22.8 months to 32.1 months. These results strongly support the integration of atezolizumab as a first-line treatment option, as it significantly improved both PFS and OS, as presented in Table 4.

The meta-analysis of four studies examining the efficacy of atezolizumab in treating cervical cancer yielded pooled estimates for progression-free survival and overall survival. The pooled median increase in PFS was 3.3 months, with a 95% confidence interval of 2.9 to 3.7 months, indicating a significant extension of PFS among patients treated with atezolizumab. For OS, the pooled median increase was 9.3 months, with a narrow 95% CI of 8.8 to 9.8 months, suggesting a substantial improvement in survival outcomes with atezolizumab treatment. However, the heterogeneity among the studies was high (I^2^ = 84%), indicating considerable variability in the treatment effects of atezolizumab across different study designs, patient populations, and treatment regimens. This level of heterogeneity suggests that while atezolizumab shows promise, its effectiveness may vary significantly under different clinical conditions or within different patient subgroups.

## 4. Discussion

### 4.1. Summary of Evidence

The analysis revealed that while atezolizumab improves median progression-free survival and overall survival in cervical cancer patients, the treatment’s efficacy varies significantly with the disease stage and previous treatment regimens. Patients in advanced stages IVB showed an OS increase from 22.8 to 32.1 months when treated with atezolizumab in combination with standard therapies. However, the high incidence of SAEs, especially in studies where intensive treatment regimens were used, raises concerns about the safety profile of atezolizumab. For instance, in the largest study reviewed, 79% of participants experienced grade ≥3 SAEs, underscoring the need for careful patient selection and monitoring.

The differential response based on prior treatments—particularly the lower SAEs in patients with extensive pretreatment (18.1% in patients with prior GOG 240 regimen)—suggests that atezolizumab might be more tolerable in patients who have already undergone rigorous cancer therapy. This is contrasted by a high rate of SAEs in previously less-treated patients, indicating that initial therapy intensity might precondition patients’ tolerance to subsequent treatments.

Moreover, the variance in disease control rates and survival outcomes across studies highlights the complex interaction between immunotherapy and host factors, such as PD-L1 expression and tumor histology. Patients with PD-L1 positive tumors showed better outcomes in the monotherapy setting, suggesting that biomarker-driven treatment strategies could enhance the efficacy of atezolizumab. This supports the potential for personalized medicine approaches in optimizing treatment plans for cervical cancer patients.

The current systematic review evidenced a significant improvement of patients’ outcomes after atezolizumab treatment. This focus on atezolizumab alone is justified given the variety of PD-1/PD-L1 inhibitors available and the differential responses observed among these therapies in cervical cancer. For instance, the CLAP trial involving camrelizumab combined with apatinib demonstrated an ORR of 55.6%, and a median PFS of 8.8 months in patients with advanced cervical cancer, showing significant promise [29]. Comparatively, pembrolizumab showed an extension in overall survival by 10.35 months and PFS by 8.50 months in a systematic review, emphasizing its efficacy particularly in patients with high PD-L1 expression [30]. Furthermore, nivolumab displayed an ORR of 48% with a six-month PFS rate of 50%, highlighting its potential in advanced disease stages [31]. These findings underscore the variable efficacy profiles of different PD-1/PD-L1 inhibitors, which justify a focused investigation into each agent, including atezolizumab, to elucidate their unique contributions to the management of cervical cancer [32]. This comparative perspective is crucial as it suggests that while many options are available, the choice of inhibitor could be critical depending on the specific patient profiles and disease characteristics. The ongoing Phase II VolATIL study [33] and the Phase III BEATcc trial [34] were not included in the current systematic review due to their statuses as active, with outcomes yet to be determined at the time of analysis. The VolATIL study is particularly promising as it explores the synergy between UCPVax, a telomerase CD4 TH1-inducer cancer vaccine, and atezolizumab in treating HPV-positive cancers. This approach aims to enhance the immunogenicity and clinical efficacy of anti-PD-L1 therapy by activating and sustaining tumor-specific T-cell responses, a strategy underpinned by preclinical data that suggest TH1 CD4 T cells are critical in supporting robust CD8+ T-cell mediated tumor rejection. On the other hand, the BEATcc trial [35] is a significant endeavor assessing the potential improvement in overall survival by adding atezolizumab to the standard cisplatin-paclitaxel and bevacizumab regimen for metastatic, recurrent, or persistent cervical cancer. Their outcomes could provide crucial data on the efficacy and safety profiles of atezolizumab in diverse therapeutic combinations, potentially setting new standards in the treatment landscape of cervical and other HPV-related cancers.

Other trials on ICIs for cervical cancer have demonstrated mixed outcomes, indicating the evolving nature of these treatments in advanced stages of the disease. Maiorano et al. [36] analyzed 17 studies showing an overall response rate from 0% to 65.9%. Particularly in PD-L1-positive patients, ORR varied from 5.9% to 69.6%, suggesting better responses in this subgroup. The disease control rate extended from 30.6% to 94.1%, while median progression-free survival ranged from 2 to 10.4 months. Median overall survival varied widely from 8 months to not reached, highlighting the potential impact of ICIs on longevity. In contrast, Kagabu’s study [37] emphasized that despite advancements with ICIs in other cancers, cervical cancer trials have remained in the phase II stages without advancing to larger phase III trials. Their analysis of trials like KEYNOTE-158 and CheckMate 358 revealed that while ICIs show promise, the median overall survival for advanced cervical cancer still sits at 16.8 months with a five-year overall survival rate of 68%, underlining the inadequate treatment outcomes and the urgent need for new therapeutic approaches. This gap suggests the potential benefit of integrating ICIs with existing therapies like concurrent chemoradiation therapy, aiming for enhanced efficacy and patient outcomes in cervical cancer.

Recent studies shed light on the nuanced application of immune checkpoint inhibitors in treating cervical cancer, particularly emphasizing the roles of PD-L1 expression and the potential for immunotherapy retreatment. Guiling Li [38] and colleagues highlighted the potential of ICI retreatment with a combination including camrelizumab, achieving an objective response rate of 26.7% and a disease control rate of 46.7%, with median progression-free survival of 3 months and overall survival of 8 months. This suggests that ICIs remain a viable option even after initial failure. Daniel Jia Ming Ang’s review [39] discusses ongoing clinical trials such as INTERLACE and KEYNOTE-A18, which are pivotal in shaping future treatment paradigms by integrating ICIs with other therapeutic modalities for both advanced and locally advanced cervical cancer. Further insights from Wutao Chen’s meta-analysis [40] indicate that in cervical cancer patients with low PD-L1 expression, ICI monotherapy was associated with adverse survival outcomes compared to ICI combination therapy or non-ICI treatments, with significant differences in OS (HR = 2.60) and PFS (HR = 7.59). These findings underscore the importance of tailored therapeutic strategies based on PD-L1 status and previous treatment outcomes, paving the way for more personalized approaches in the management of cervical cancer.

Recent studies exploring the combination of immune checkpoint inhibitors with other agents have shown varying degrees of success in treating advanced solid tumors. Sherman et al. [41] evaluated the efficacy of combining cobimetinib, a MEK inhibitor, with atezolizumab, demonstrating moderate activity in treatment-naive squamous cell carcinoma of the head and neck and urothelial carcinoma with objective response rates of 20% and 30%, respectively. However, this combination showed limited effectiveness in renal cell carcinoma and no activity in previously treated squamous cell carcinoma of the head and neck patients. Meanwhile, Simonelli et al. [42] assessed isatuximab, an anti-CD38 antibody, with atezolizumab, revealing that while the combination was well tolerated and effectively engaged its target by reducing CD38+ immune cells in the tumor microenvironment, it failed to enhance responsiveness to the PD-L1 blockade, leading to discontinuation of the trial in its second stage.

Recent phase I studies have highlighted the evolving landscape of combination immunotherapies in the treatment of advanced solid tumors, specifically emphasizing the role of novel checkpoint inhibitors alongside established therapies. The YP42514 study [43] assessed tiragolumab combined with atezolizumab, revealing that this regimen was generally well tolerated with a manageable safety profile, achieving partial responses in 10% of the Chinese cohort with advanced solid tumors, predominantly non-small cell lung cancer. Similarly, the GO30103 trial [44] evaluated tiragolumab’s safety and preliminary efficacy, identifying a recommended phase 2 dosage and demonstrating encouraging antitumor activity, particularly in immunotherapy-naive patients with non-small cell lung cancer and esophageal cancer, achieving objective response rates of 46% and 28%, respectively. These studies not only reinforce the potential of combining TIGIT inhibitors with PD-L1 inhibitors in enhancing anticancer responses but also underscore the need for further investigation into optimal dosing strategies and patient selection to maximize therapeutic outcomes. 

The critical analysis of recent phase 3 trials examining PD-1/PD-L1 inhibitors for cervical cancer reveals nuanced findings regarding their efficacy and safety profiles. In the CALLA trial, durvalumab added to chemoradiotherapy did not significantly improve progression-free survival, with both treatment groups (durvalumab and placebo) not reaching a median PFS at 18.5 months follow-up; however, the study highlighted durvalumab’s safety, suggesting that further investigation in PD-L1 biomarker-selected populations might be warranted [45]. Conversely, the EMPOWER-Cervical 1/GOG-3016/ENGOT-cx9 trial reported that cemiplimab significantly extended median overall survival to 12.0 months compared to 8.5 months with chemotherapy, demonstrating a clear benefit in recurrent cervical cancer [46]. Pembrolizumab, in the KEYNOTE-826 trial, showed improved PFS and overall survival when added to chemotherapy, with a notable survival advantage in patients with a PD-L1 combined positive score of 10 or more, indicating its efficacy in a more targeted patient group [47]. 

The differentiation between HPV-positive and HPV-negative cervical cancers is crucial, particularly when considering immune checkpoint inhibitors that rely on reactivating immune responses, as outlined in recent studies. Zhang et al. [48] emphasize that HPV-positive cervical cancers often have an increased expression of PD-L1/PD-1 due to HPV oncogene-induced activation of immune evasion pathways, providing a rationale for targeted blockade therapies in these cases. Conversely, the two studies by Evans et al. [49,50] highlight that HPV-negative cancers exhibit distinct immunological landscapes, characterized by lower lymphocyte infiltration and reduced expression of MHC class I and II, suggesting a potentially reduced efficacy of ICIs in these subtypes. 

The distinction between squamous cell carcinoma and adenocarcinoma in cervical cancer significantly impacts PD-L1 expression and immunotherapy outcomes. Heeren et al. [51] found SCC more frequently associated with PD-L1 expression, correlating with poorer disease-specific outcomes, particularly when PD-L1 is diffusely expressed. PD-L1-positive tumor-associated macrophages in ACC were linked to worsened survival, suggesting complex interactions between tumor microenvironment and histological type. Conversely, Hosseini et al. [52] observed higher, though non-significant, PD-L1 expression in SCC over ACC, with no strong correlation to clinical characteristics in either subtype. These findings underline the necessity of considering both histological subtype and PD-L1 status in the personalized immunotherapeutic approach for cervical cancer, supporting the integration of detailed immunohistochemical profiling in treatment planning.

Overall, this systematic review contributes significantly to the understanding of atezolizumab as a therapeutic option for cervical cancer, specifically highlighting its potential to improve PFS and OS when integrated with standard treatments. The study brings evidence from randomized clinical trials showing that atezolizumab, when added to standard therapies or used as monotherapy, can extend median PFS with more than 10 months and OS with more than 20 months. This suggests that atezolizumab could be a viable first-line treatment option for patients, particularly in advanced stages of cervical cancer. This review also provides critical insights into the safety profile of atezolizumab, noting a high incidence of serious adverse events which peaked in more intensive treatment combinations. 

### 4.2. Limitations

This systematic review is limited by the variability in study designs, patient populations, and treatment regimens, which may influence the generalizability of the results. Moreover, the small number of studies included may limit the generalizability of the findings. With only four studies reviewed, the range of patient demographics and treatment settings is restricted, which may not adequately reflect the broader population of cervical cancer patients treated with atezolizumab. Furthermore, the heterogeneity in the reporting of safety data, particularly serious adverse events, and different follow-up durations across studies could affect the comparative analysis of the safety profiles. This heterogeneity is particularly evident in the range of reported serious adverse events, which varied widely from 3.6% to 79%, possibly reflecting differences in reporting standards or patient populations across studies. Furthermore, the absence of a comparison group in one of the studies (Tabernero et al.) makes it difficult to contextualize atezolizumab’s efficacy relative to standard treatments or other therapies. This lack of comparative data could skew perceptions of the drug’s effectiveness and safety profile. Lastly, the varying lengths of follow-up among the studies, ranging from 9 weeks to nearly 33 months, might influence the observed outcomes, particularly in terms of long-term efficacy and safety, which are crucial for fully understanding the treatment’s impact.

## 5. Conclusions

Atezolizumab presents a promising therapeutic option for cervical cancer, offering significant improvements in survival outcomes for patients, particularly when used in combination with existing therapies. However, the benefits of atezolizumab must be weighed against its safety risks, as evidenced by the high rates of SAEs associated with its use. Future clinical trials should focus on identifying patient subgroups that are most likely to benefit from atezolizumab, based on biomarkers and prior treatment history, to maximize efficacy while minimizing adverse effects. The findings advocate for the integration of atezolizumab into standard cervical cancer treatment protocols, contingent on further validation of its safety profile and long-term benefits.

## Figures and Tables

**Table 1:** Study characteristics.

Study and Author	Country	Study Year	Study Design	Study Quality
1 [24] Friedman et al.	International	2020	Randomized clinical trial (phase II)	High
2 [25] Tabernero et al.	International	2022	Randomized clinical trial (phase II)	High
3 [26] Mayadev et al.	International	2022	Randomized clinical trial (phase I/Ib)	High
4 [27] Oaknin et al.	International	2024	Randomized clinical trial (phase III)	High

**Table 2:** Characteristics of patients.

Study and Author	Sample Size	Age (Years)	Comparison Group	Performance Status	Time Since Diagnosis
1 [24] Friedman et al.	11	Median: 48	Atezolizumab + Bevacizumab	ECOG 0: 64% ECOG 1: 36%	NR
2 [25] Tabernero et al.	27	Median: 49	No comparison	ECOG 0: 40.7% ECOG 1: 59.3%	Time since diagnosis: 3.47 years Time since metastasis: 1.52 years
3 [26] Mayadev et al.	40	Median: 48	Atezolizumab + standard chemoradiation	ECOG < 2: 100%	Newly diagnosed
4 [27] Oaknin et al.	206	Median: 51	Atezolizumab + bevacizumab + chemotherapy vs. bevacizumab + chemotherapy (n = 204)	ECOG 0: 67% and ECOG 1: 33%	Newly diagnosed stage IVB: 21% Recurrent disease: 73% Persistent disease: 6%

NR—not reported; ECOG—Eastern Cooperative Oncology Group.

**Table 3:** Disease characteristics.

Study Number	Stage	Histology	HPV/PDL1 Status	Prior Treatment	SAEs
1 [24] Friedman et al.	IVA-IVB: 100%	SCC: 55%, ACC 45%	NR	73% prior pelvic radiation with concurrent cisplatin; all had prior GOG 240 regimen	SAE (grade ≥ 3): 2 (18.1%)
2 [25] Tabernero et al.	IVA: 3.7% IVB: 96.3%	SCC: 51.9%, ACC 44.4%, moderate/poorly differentiated: 70.4%	PDL1: 77.8%	70.4% with 2 anticancer systemic treatments Radiotherapy: 92.6% Surgery: 66.7%	SAE (grade ≥ 3): 3.6%
3 [26] Mayadev et al.	IIB: 100%	NR	NR	77.5% prior brachytherapy	SAE (grade ≥ 3): 10 (25%)
4 [27] Oaknin et al.	I: 15% II: 30% III (all types): 18% IVA: 5% IVB: 21%	SCC: 80%, ACC 17%	NR	34% concurrent chemotherapy 4% surgery 31% surgery + chemotherapy	SAE (grade ≥ 3): 161 (79%)

NR—not reported; HPV—Human Papilloma Virus; PD-L1—Programmed Death Ligand; SCC—Squamous Cell Carcinoma; ACC—Adenocarcinoma; SAE—Serious Adverse Events.

**Table 4:** Analysis of outcomes.

Study Number	Treatment/Dose	Follow-Up	Outcomes/Survival	Conclusions
1 [24] Friedman et al.	Atezolizumab 1200 mg IV every 3 weeks and Bevacizumab 15 mg/kg IV every 3 weeks	Median 9 weeks	ORR: 0%; DCR: 60%; Median PFS: 2.9 months; Median OS: 8.9 months	The combination did not meet efficacy endpoints; no RECIST V.1.1 responses in the evaluable patients; two unconfirmed PRs were transient.
2 [25] Tabernero et al.	Atezolizumab 1200 mg IV every 3 weeks until loss of clinical benefit or unacceptable toxicity	18 weeks	PFS: 44.4% ORR: 14.8%	Atezolizumab monotherapy was effective in the cervical cancer cohort.
3 [26] Mayadev et al.	Atezolizumab 1200 mg IV every 3 weeks	Median 20 months	24 months DFS: 79% with atezolizumab prior to and concurrently with combined chemoradiotherapy vs. 52% concurrently with chemoradiotherapy *	Atezolizumab as a primer and concurrent with chemoradiotherapy is safe and shows immune-modulating activity in women with locally advanced cervical cancer.
4 [27] Oaknin et al.	Atezolizumab 1200 mg IV every 3 weeks (median treatment duration of 6 cycles)	Median 32.9 months	PFS: 13.7 months with atezolizumab vs. 10.4 months with standard therapy; OS: 32.1 months vs. 22.8 months	Atezolizumab addition improves PFS and OS, should be considered as a new first-line treatment option

OS—overall survival; DCR—disease control rate; PFS—progression free survival; IV—intravenous; *—NRGGY017 trial reports [28].

## Data Availability

Not applicable.
